# Repeated Task Exposure and Sufficient Sleep May Mitigate ADHD-Related Cognitive Flexibility Impairments in Family Dogs

**DOI:** 10.3390/ani15213074

**Published:** 2025-10-23

**Authors:** Tímea Kovács, Vivien Reicher, Barbara Csibra, Melitta Csepregi, Kíra Kristóf, Márta Gácsi

**Affiliations:** 1 Department of Ethology, ELTE Eötvös Loránd University, Pázmány Péter Sétány 1/c, 1117 Budapest, Hungary; 2 Doctoral School of Biology, ELTE Eötvös Loránd University, Pázmány Péter Sétány 1/c, 1117 Budapest, Hungary; 3 Clinical and Developmental Neuropsychology Research Group, Institute of Cognitive Neuroscience and Psychology, Research Centre for Natural Sciences, Magyar Tudósok Körútja 2, 1117 Budapest, Hungary; 4 Department of Psychiatry and Psychotherapy, Semmelweis University, Balassa Utca 6, 1083 Budapest, Hungary; 5 HUN-REN–ELTE Comparative Ethology Research Group, Pázmány Péter Sétány 1/c, 1117 Budapest, Hungary

**Keywords:** family dog, ADHD, cognitive flexibility, reversal learning, sleep

## Abstract

**Simple Summary:**

Family dogs can exhibit symptoms resembling Attention-Deficit/Hyperactivity Disorder (ADHD). This study explored whether ADHD-like traits in dogs, as in humans, are associated with impaired cognitive flexibility, and how sleep may influence this relationship. We tested 64 family dogs in a reversal learning task: first, they learned to identify which of two pots was baited with food (discrimination phase), after which the rewarded side was switched (reversal phase). Following sleep electroencephalography (EEG) measurement, the task was repeated. Owners completed a validated questionnaire to rate ADHD-like behaviours in their dogs. Dogs with stronger ADHD-like traits learned slower the first reversal, but not after sleep. Electrode application was slower and the sleep measurement was more likely to fail in dogs with higher ADHD scores; however, their reversal learning performance improved more from pre- to post-sleep if they slept for at least 25 min. These findings parallel human research linking ADHD symptoms to reduced cognitive flexibility and further suggest that repetition and sufficient sleep may mitigate such impairments. Cognitive training may therefore represent a promising strategy to support dogs with ADHD-like behaviours.

**Abstract:**

The family dog is a valid model for studying complex human functions and psychological disorders such as Attention-Deficit/Hyperactivity Disorder (ADHD). Based on prior human research indicating impairments in cognitive flexibility related to ADHD, this study investigates the association between dogs’ ADHD-like traits and reversal learning performance. Since sleep improves learning both in humans and dogs, we also examined its impact in this context. Family dogs (N = 64) completed a two-way choice spatial reversal learning task, followed by a one-hour non-invasive sleep electroencephalography (EEG), and then a second reversal task. We used a validated human analogue questionnaire to assess ADHD. Dogs with higher ADHD scores required more trials to pass the first reversal test, but not after sleep. Electrode application was slower and sleep measurement more likely to fail in dogs with higher ADHD scores. Performance improved more from pre- to post-sleep in high-ADHD dogs if they spent at least 40% of the recording asleep. Our findings align with the human literature showing associations between ADHD and cognitive flexibility in dogs. The main novelty here is the ADHD-related potential benefits of repeated task exposure after sufficient sleep on cognitive flexibility. Cognitive training offers a promising direction to mitigate ADHD-related impairments in dogs.

## 1. Introduction

Cognitive flexibility, the ability to adjust responses to changing environmental demands, is essential for adaptive behaviour in both humans and non-human animals [1,2]. In animals, it has been linked to improved survival, reproductive success [3], and effective social functioning, particularly in managing complex interactions with conspecifics [4]. In humans, greater cognitive flexibility has been associated with higher academic achievement [5], and it has a protective role in coping with stressful life events [6]. In contrast, cognitive rigidity has been observed as part of the cognitive decline associated with ageing [7] and has been linked to maladaptive behaviours such as self-injury [8] and suicidal ideation [9,10]. Impairments in cognitive flexibility are also evident in neurodevelopmental disorders such as Attention-Deficit/Hyperactivity Disorder (ADHD) [11]. Both children and adults with ADHD showed worse performance compared to peers without ADHD in tests measuring cognitive flexibility, like the Wisconsin Card Sorting Test and a set-shifting task [12,13]. The broad and far-reaching impact of cognitive flexibility on everyday functioning underscores the importance of understanding its mechanisms, which can be explored through animal research as well.

Family dogs (*Canis familiaris*) have long served as valuable subjects in comparative research modelling human functions [14,15,16] such as cognitive flexibility. While most tests of cognitive flexibility are suitable only for human research, the reversal learning paradigm is a straightforward method that can be applied in several animal species, including dogs [17]. In this task, subjects first learn to discriminate between two or more stimuli, one of which is rewarded. Then the reward contingency is switched to another stimulus, requiring them to adapt their behaviour accordingly. Prior canine research focusing on ageing-related cognitive decline has found that, similarly to humans, reversal learning performance declines with age [18,19,20,21]. However, most existing studies rely on one-time testing, and we argue that repeated assessments may offer deeper insights into the stability of cognitive flexibility. Supporting this view, a longitudinal study in children reported some improvements over time [22], and short-term practice effects have been observed in adolescents [23]. Furthermore, repetition-based interventions are becoming recognised as promising tools for the treatment of ADHD symptoms [24,25].

Family dogs are also valuable animal model species for ADHD research. ADHD-like traits such as inattention, hyperactivity, and impulsivity naturally emerge in dogs [26,27,28,29] and genetic studies identified candidate genes which are also implicated in human ADHD [30,31,32]. These traits can be measured using a validated owner-report questionnaire that assesses all three ADHD dimensions (inattention, hyperactivity, impulsivity) [33] and has been validated through behaviour testing [34]. Although formal diagnostic criteria for canine ADHD have only recently been introduced [35], previous dog research has already linked ADHD-like traits to demographic [26,29,33,36,37] and personality characteristics [37], as well as to sleep [38] and learning [39,40,41], reporting findings that closely resemble those observed in human ADHD research.

While both cognitive flexibility and ADHD-like traits have been studied in dogs, their relationship remains unexplored. Given that impairments in cognitive flexibility are well-documented in humans with ADHD, investigating this link in dogs is a relevant next step. Our study aimed to examine the association between ADHD-like traits and cognitive flexibility in family dogs using a reversal learning paradigm. Since sleep has been shown to enhance learning performance in dogs [42,43], we conducted a second reversal learning test following a 1-h sleep session monitored with non-invasive electroencephalography (EEG). This method, which requires no pre-training [44], enables the investigation of the impact of sleep on learning performance, a phenomenon well-established across species [45].

We hypothesised that dogs with higher ADHD scores would perform worse in reversal learning, requiring more trials to reach the learning criterion. Alternatively, if cognitive flexibility is not a stable trait but can improve through experience and/or sufficient sleep, we expected these performance differences to appear in the first test and not in the post-sleep test. Regarding the sleep EEG measurement, we expected that in dogs with higher ADHD scores, electrode attachment would be less successful and/or take longer, given that the procedure depends on the dog’s ability to remain calm and still.

## 2. Materials and Methods

### 2.1. Ethics Statement

**Figure 1:**
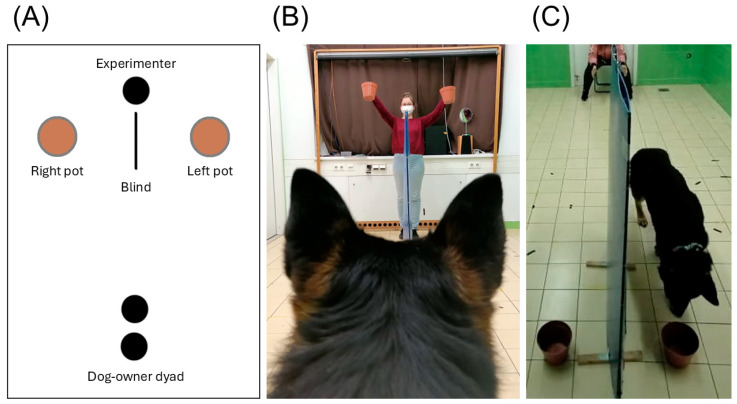
The experimental setup. (**A**) A schematic overview of the arrangement. (**B**) The owner’s point of view. The experimenter holds up the pots before simultaneously placing them on the ground. (**C**) The experimenter’s point of view. The dog is released by the owner and approaches one of the baited pots.

The experimental protocol received ethical approval from the Animal Welfare Committee of Eötvös Loránd University (PE/EA/00035-4/2023). All methods were carried out following relevant guidelines (including ARRIVE) and regulations. The test was performed following the EU Directive 2010/63/EU and the recommendations of the Hungarian State Health and Medical Service. The questionnaire data collection was approved by the United Ethical Review Committee for Research in Psychology (EPKEB; Ref. no.: 2023-04). Before testing, owners were informed about the circumstances of the measurements and signed a written consent. The experimenter shown in Figure 1B provided written consent for the use of her photograph.

### 2.2. Subjects

Subjects were recruited via popular social media platforms, the Family Dog Project participant pool, and snowball sampling. First, owners filled out an online questionnaire about the dogs’ demographic data and ADHD-like traits (see Section 2.3. Questionnaire). Second, owners were invited based on their reports, covering the range of the ADHD score scale as large as possible. Aiming for a normal distribution of ADHD scores and considering the positively skewed distribution in the population [33], we specifically invited higher-scoring individuals (see Figure S1 for the distribution in our sample). Participation was voluntary, and owners could stop the test at any time.

We included 64 dogs in the test. Due to experimenter error (i.e., conducting more than three extra unnecessary trials or terminating a test although the subject reached the learning criterion), we excluded 6 dogs. The final sample of 58 dogs consisted of 29 males and 29 females (21 males and 24 females were neutered), aged 0.8–8.6 years (M = 3.6, SD = 2.1 years) from 20 different breeds and 23 dogs were mutts. Since age, sex, and training level have been shown to be associated with ADHD traits [33], we balanced the sample for these variables and accounted for them in the analyses.

Sleep EEG data collection was attempted in a subset of 46 dogs from the total sample, as the other owners declined participation due to their limited availability.

### 2.3. Questionnaire

Besides providing basic demographic data, owners completed the Dog ADHD and Functionality Rating Scale (DAFRS) questionnaire [33] prior to testing. This human-analogue questionnaire allows for the measurement of the dog’s inattention, hyperactivity, and impulsivity, as well as related functional impairments. Owners responded on 4-point Likert-scales on how often a certain behaviour occurs (0 = never, 1 = sometimes, 2 = often, and 3 = very often). Example items include “has difficulties concentrating” (inattention), “fidgets, bustles” (hyperactivity), and “has no self-control” (impulsivity). The total ADHD scores of dogs were calculated based on the methods described in the study of Csibra et al. [35].

### 2.4. Experimental Setup

The tests took place at the Department of Ethology, Eötvös Loránd University, Budapest, Hungary. In order to avoid the first-night effect [46], an adaptation sleep session (1 h duration) was conducted approximately one week prior to the test session in dogs who had not recently (within a year) participated in a sleep EEG study and/or did not frequently sleep away from home (known to affect the sleep macrostructure in dogs [46]). In the test occasion, dogs participated in a reversal learning task (typically 20–30 min, depending on the number of trials needed), a 1-h-long sleep measurement, and again the same reversal learning task. All test occasions took place in the afternoon to reduce potential variability related to circadian rhythm influences. The behaviour laboratory was equipped with a camera system. Sleep measurements were conducted in a sleep laboratory fully equipped for non-invasive canine EEG measurements. A mattress and a reading lamp were provided for the comfort of the owner and the dog, creating a calm, dark and quiet environment for the dog to settle and fall asleep, while the experimenter controlled the data acquisition from outside the laboratory (see details in [46]).

### 2.5. Procedure

#### 2.5.1. General Procedure

Before the discrimination and reversal learning test, a short, 10-min-long behaviour test battery was conducted, the results of which were analysed and published separately. For more information, see [34].

We used a two-way choice spatial reversal learning paradigm; a piece of treat was placed in one of two pots positioned 1 m apart and separated by a blind. During the test, the owner was sitting on a chair along the laboratory’s midline, 3 m away from the blind and baited pots. See Figure 1 for a schematic overview and photos of the experimental setup.

Before the two-way choice, to ensure that the dog associated the pots with food, we conducted an encouraging trial with one pot placed in the middle. The experimenter (E) attracted the dog’s attention by calling its name and placed a treat in the pot. The owner then released the dog, allowing it to eat the treat. Next, a one-trial preference test with both pots baited was conducted to assess any existing side preference (e.g., [47,48]). To avoid reinforcing the potential side preference, the baited side in the pre-sleep discrimination learning phase (d1) was set to the opposite of the dog’s choice in the preference test. This design helped prevent a confounding effect of strong side preference (indeed, two subjects that did not pass the d1 phase were excluded because they consistently chose the incorrect side in all d1 trials).

**Figure 2:**
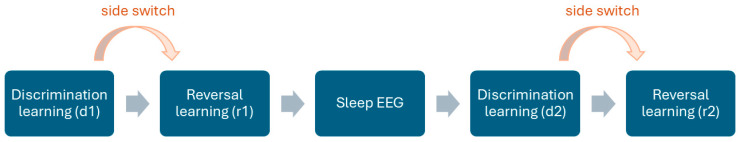
The phases of the reversal learning paradigm. Phase d1 was preceded by a short behaviour test battery [34], an encouraging trial, and a preference test. Reward contingencies were switched after phases d1 and d2, while the baited side in d2 remained the same as in r1. See the subsample sizes in Table S1. The arrows depict the chronological order of the phases.

The structure of the test is summarised in Figure 2.

#### 2.5.2. Discrimination and Reversal Learning

In the discrimination and reversal learning phases, the dog was off-leash, held by a collar or harness, and released by the owner right after E simultaneously placed both pots on the ground. If the dog did not move after being physically released, the owner provided a release command for encouragement. Once the dog reached one of the pots, E lifted the other immediately to prevent the dog from accessing both in the same trial.

At the start of each trial, the owner held the dog’s collar or harness while E baited the predetermined side (based on the preference test). To prevent the dog from seeing the baiting process, E stood with her back to the dog. The dog had up to 5 s to make a choice and approach one side (one of the pots), while E maintained a neutral posture, looking straight ahead without making eye contact. The trial ended (1) once the dog reached one of the pots and ate the food, or (2) 5 s elapsed without the dog approaching either pot. The owner was instructed to remain passive during the trials and was not allowed to encourage the dog, but could recall and praise the dog between trials. Each trial was live-coded as correct, incorrect, or no-choice (if the dog did not approach either pot within the time limit). No-choice trials were followed by an encouraging trial (similar to the first encouraging trial), where one pot was placed in the centre. During the encouraging trial, the owner was allowed to encourage the dog (e.g., by saying ‘Search it!’ or pointing in the direction of the pot). These encouraging trials were not included in the analyses. If the dog had three (not necessarily consecutive) no-choice trials, the test and data collection was terminated. To pass the test, the dog had to have 8 correct choices within the last 10 trials (excluding encouraging trials). Dogs completed a minimum of 10 and a maximum of 40 trials in all discrimination and reversal learning phases. Those that did not reach the learning criterion in 40 trials were not tested in the next learning phase.

If the dog completed the discrimination phase, dogs were given a short break of 3–5 min before starting the reversal learning phase. During this break, they were offered water. The reversal learning phases (r1, r2) followed the same procedure as the discrimination phases, with the exception that the previously baited side was switched.

#### 2.5.3. Sleep EEG

Sleep measurements were conducted by two experienced researchers (V. R. and B. C.) to ensure that the success of electrode attachment was not dependent on the experimenter’s skill. A sleep measurement was considered successful if (i) the initial electrode attachment procedure could be carried out within 45 min, (ii) the dog fell asleep within 30 min after the start of the recording, and (iii) at least three functioning electrodes (G1, G2, and at least one more) sufficient for analysis remained attached. If one or more electrodes were detached by the dog’s movement, the experimenter reattached them. In this study, electrode attachment failed in some cases due to the dog’s restlessness (extensive fidgeting) or aggressive behaviour. For unsuccessful measurements, the between-test interval was always extended to at least one hour to reduce variability between dogs with successful and unsuccessful recordings. Sleep measurement recordings started once all electrodes were successfully attached and stopped after 1 h.

We used a previously validated protocol for dogs to monitor sleep through electroencephalography (EEG) and electrooculogram (EOG) [44,46]. The two electrodes placed on the right and left zygomatic arch next to the eyes (F8, F7) and the scalp electrodes over the anteroposterior midline of the skull (Fz, Cz) were referred to the G2, a reference electrode which was in the posterior midline of the skull (occiput; external occipital protuberance). The ground electrode (G1) was attached to the left musculus temporalis. Gold-coated Ag/AgCl electrodes were used for the recordings and secured with Signa Spray Electrode Solution (Parker, Fairfield, NJ, United States) and EC2 Grass Electrode Cream (Grass Technologies, West Warwick, RI, United States). The owner was asked to stay quiet and sit still next to their dog during the measurements, while E monitored the recordings from outside the sleep laboratory via a computer.

The signal was collected, pre-filtered, amplified, and digitised at a sampling rate of 1024 Hz per channel using a SAM 25 R-style MicroMed Headbox (MicroMed Inc., Houston, TX, USA). The hardware passband was set between 0.5 and 256 Hz, with a sampling rate of 512 Hz, an anti-aliasing filter with a cutoff frequency of 1 kHz, and 12-bit resolution covering a voltage range of ±2 mV. Additionally, second-order software filters were applied using System Plus Evolution software (MicroMed Inc., Houston, TX, USA), including a high-pass filter (>0.016 Hz) and a low-pass filter (<70 Hz).

### 2.6. Data Analysis

Trial outcomes were live-coded during the tests. As performance indicators, we analysed the number of pre-sleep and post-sleep reversal trials (r1 and r2, respectively) required to reach the learning criterion. To assess performance improvement, we calculated the difference in trial counts between r2 and r1 by subtracting the r1 trial count from r2.

The time needed to attach the electrodes was recorded as follows: after the 5-min exploration period in the sleep laboratory, E started the attachment procedure and a stopwatch at the same time. The stopwatch was stopped once the electrodes were in place and recording began. If any electrodes detached during the measurement and had to be reattached, the extra time was added to the total. However, the maximum total time for electrode attachment was limited to 45 min.

Sleep recordings were visually scored (by T. K.) in a self-developed programme (by Ferenc Gombos; Fercio’s EEG Plus, 2009—2023), following standard criteria [49] adapted for dogs [44]. For inter-rater reliability, another researcher (V. R.) also scored 4 dog measurements (180 epochs/recording). Reliability was very high (Cohen’s κ = 0.74). This manual scoring method reliably distinguishes between wakefulness, drowsiness, NREM, and REM sleep in dogs [50], enabling the identification of sleep latency, which was necessary for evaluating whether a sleep recording was considered successful by definition. Sleep efficiency was also calculated (percentage of time spent asleep: drowsiness + NREM + REM). We focused exclusively on this parameter due to the relatively short duration of the measurement, and our aim to obtain a general overview of the dogs’ sleep quality. Further, prior research highlighted its relevance in learning contexts [43].

### 2.7. Statistical Analysis

To analyse the performance data (three variables: r1 and r2 trial counts, and r2-r1 trial count difference), Generalised Linear Models (GLMs) were used. Normality of the dependent variables was checked with Shapiro–Wilk normality tests. Two of the dependent variables (r1 trial count and r2 trial count) followed a Poisson distribution, while the r2-r1 trial count difference followed normal distribution. Thus, the models were built accordingly. The included independent variables for r1 and r2 trial counts were total ADHD score, training level, and age. For performance improvement (r2-r1), the included variables were the interaction of total ADHD score × sleep efficiency, training level, and age. Note that in this sample, all dogs that passed phase r2 were included (dogs with an unsuccessful EEG measurement were included with a sleep efficiency of 0%). R^2^ values were calculated for each model as an estimate of effect size. For post hoc analysis of the categorical variable training level, estimated marginal means were compared, whereas for the interaction of two continuous variables (total ADHD score × sleep efficiency), the Johnson-Neyman technique [51] was used with false discovery rate correction. This method allows the identification of the range of moderator values where the predictor’s effect on the response is significant. No multicollinearity was detected among the predictors in any of the models.

The time needed to attach the electrodes was analysed with a Cox proportional hazards model, which is well-suited for examining whether and when an event occurred, i.e., censored outcomes. The included independent variables were the total ADHD score, training level, and age. No multicollinearity was detected among the predictors.

The success of the sleep measurement was analysed with a binomial Generalised Linear Model with independent variables of the total ADHD score, training level, and age. No multicollinearity was detected among the predictors.

The statistical analyses and visualisation of the results were conducted in the software R Studio (version 4.4.1) [52]. The following functions were used; ‘descdist’ function of ‘fitdistrplus’ package [53] for identifying non-normal distributions, ‘glm’ function of ‘stats’ package for GLMs, ‘coxph’ function of ‘survival’ package [54] for the Cox proportional hazards model, ‘check_model’ function of ‘performance’ package [55] for checking model assumptions, ‘emmeans’ function of ‘emmeans’ package [56] for post hoc comparisons, ‘r2’ function of ‘performance’ package [55] for effect size estimation, and lastly, ‘ggplot’ function of ‘ggplot2’ package [57], ‘survfit’ function of ‘survival’ package [54], and ‘ggsurvplot’ function of ‘survminer’ package [58] for visualisation.

## 3. Results

For an overview of the results of the GLM analyses of the associations between total ADHD score, training level, age, sleep efficiency (where applicable) and performance outcomes, as well as the success of the sleep EEG measurements, see Table S2.

### 3.1. Performance

Demographic data of the dogs that passed/failed the pre- and post-sleep discrimination and reversal learning phases, i.e., whether they reached the learning criterion, are summarised in Table S1.

**Figure 3:**
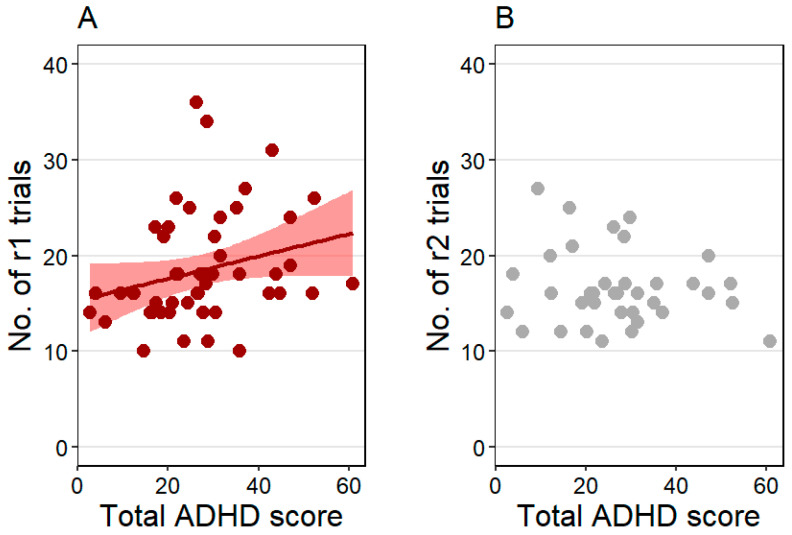
(**A**) The positive association between the total Attention-Deficit/Hyperactivity Disorder (ADHD) score and the pre-sleep reversal (r1) trial count and (**B**) no association between the total ADHD score and the post-sleep reversal (r2) trial count. Individual data points and, for the significant effect, the regression line with SE are shown for clearer illustration.

The number of pre-sleep reversal trials (r1) was positively associated with the total ADHD score (z = 2.75, *p* = 0.006), indicating that dogs with higher total ADHD scores needed more reversal trials to reach the learning criterion (Figure 3A). Training level and age were not associated with the pre-sleep reversal trial count. The effect size was R^2^ = 0.24 in this model.

The number of post-sleep reversal trials (r2) was not associated with the total ADHD score (Figure 3B) and training level, but was positively linked to age (z = 2.13, *p* = 0.033), showing that older dogs needed more post-sleep reversal trials to reach the learning criterion. The effect size was R^2^ = 0.29 in this model.

### 3.2. Sleep

**Figure 4:**
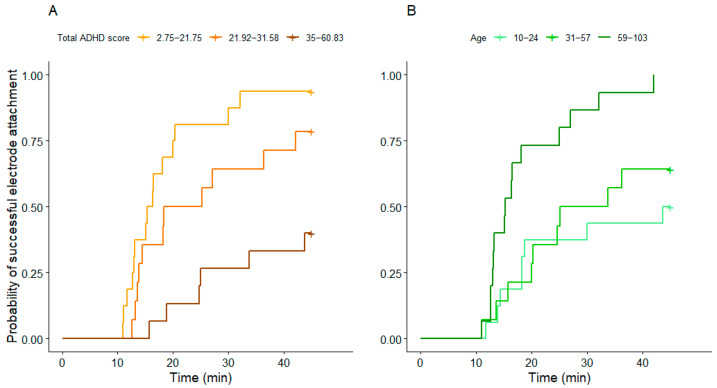
The association between (**A**) the total ADHD score and (**B**) age with the time needed for electrode attachment. Both predictors were entered as continuous variables in the model; they are grouped into tertiles for visualisation purposes only. Age is shown in months.

The time needed to attach the electrodes was associated with total ADHD score and age, but not with training level. Electrode attachment took longer in dogs with higher total ADHD scores (z = −3.18, *p* = 0.001) (Figure 4A) and in younger dogs (z = 2.18, *p* = 0.029) (Figure 4B). The results of the Cox proportional hazards model are summarised in Table S3. The effect size was R^2^ = 0.41 in this model.

**Figure 5:**
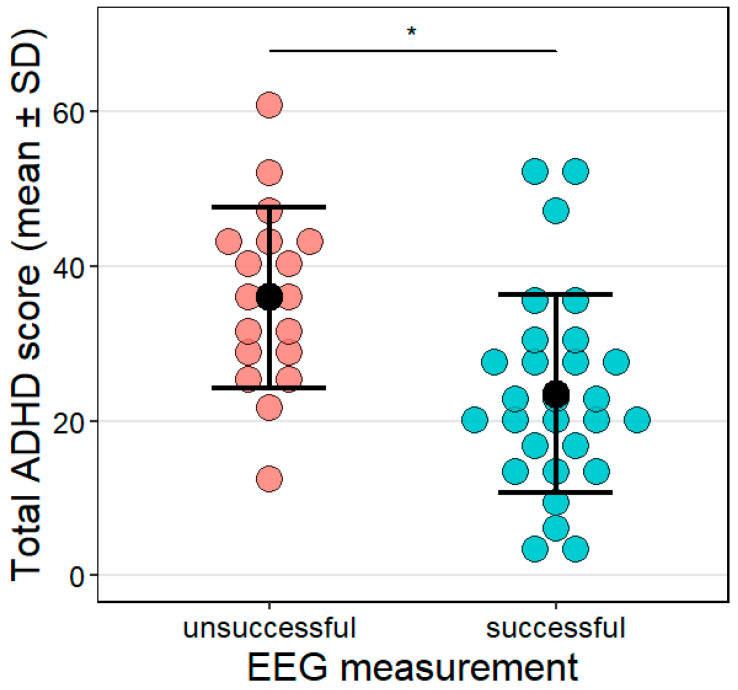
The link between the total ADHD score and the success of the sleep EEG measurement. Success was defined based on the criteria described in Section 2.5.3. Sleep EEG. Individual data points as well as the mean (black dot) and SD are shown for the groups. * *p* < 0.05.

Sleep EEG measurements were successful in 60.87% of the sample (N = 28; total ADHD score: M = 23.5, SD = 12.87). The other 18 dogs were excluded from the sleep analysis (total ADHD score: M = 35.99, SD = 11.7); for 13 dogs, electrode attachment could not be carried out within the pre-set timeframe, and 5 dogs did not fall asleep within 30 min from the start of the EEG recording, thus their measurement was terminated. Dogs whose EEG measurements were unsuccessful had higher total ADHD scores than those that could successfully be measured (z = −2.3, *p* = 0.021, Figure 5). The effect size was R^2^ = 0.29 in this model.

### 3.3. Performance Improvement and Sleep

**Figure 6:**
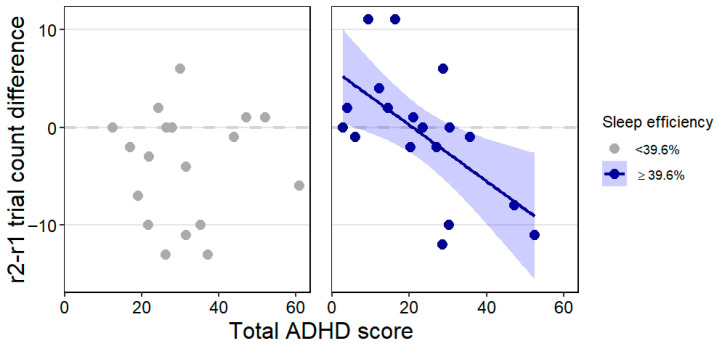
The trend-level association between the total ADHD score × sleep efficiency interaction and performance improvement (r2-r1 trial count difference) highlighting the range in which the association is statistically significant. Minus values on the *y*-axis indicate performance improvement (decrease in the number of trials needed to reach the learning criterion). Individual data points and, for the significant association, the regression line with SE are shown for clearer illustration.

Since performance improvement (pre- to post-sleep reversal trial count difference) showed a trend-level association with the interaction of total ADHD score and sleep efficiency, we applied post hoc Johnson-Neyman analysis which revealed a statistically significant (*p* < 0.05) association in dogs with a minimum of 39.6% sleep efficiency. That is, in dogs who spent at least approximately 40% of the recording time sleeping, higher total ADHD score was associated with greater performance improvement (lower r2-r1 trial count difference value, see Figure 6). This association was not observable in dogs who spent less than 40% of the recording asleep (or could not be measured with the EEG procedure, thus did not sleep and were not recorded at all). Training level and age were not associated with performance improvement. The effect size was R^2^ = 0.34 in this model.

## 4. Discussion

To investigate factors influencing cognitive flexibility in family dogs, we examined how owner-rated ADHD-like traits relate to performance in a reversal learning task. By conducting repeated testing, we also assessed the stability of cognitive flexibility in this respect and the potential role of the amount of sleep in improving learning performance. Our findings indicated that dogs with higher ADHD scores needed more reversal trials to reach the learning criterion in the first but not in the second test. Importantly, dogs with higher ADHD scores who obtained sufficient sleep between the two tests showed greater performance improvement. We also revealed associations between dogs’ ADHD scores and the feasibility of a non-invasive sleep EEG methodology.

Consistent with our hypothesis, higher ADHD scores (a total score summing inattention, hyperactivity and impulsivity subscale scores) were associated with poorer performance in the first reversal learning task, mirroring results reported in human studies [59,60,61]. This pattern also aligns with findings from dog–wolf comparisons; dogs (raised similarly to wolves) showed greater flexibility and outperformed wolves in a reversal learning task [62]. Given that dogs exhibit better inhibitory control abilities than wolves [63,64] and that impaired inhibition has also been linked to ADHD-like traits in dogs [34,40,65], this result is in line with expectations.

However, there was no relationship between ADHD score and reversal learning performance in the second, post-sleep test. One possible explanation is that repeated task exposure, that is, experience in the reversal paradigm, selectively benefited dogs with higher ADHD scores. Supporting this, previous findings indicated that more inattentive dogs performed better in a command-learning task with repetitive training [41]. In humans, cognitive flexibility training has shown effectiveness in children with Autism Spectrum Disorder [66], though similar interventions did not improve flexibility in children with ADHD in a pilot study [67]. Nonetheless, some evidence suggests that psychosocial interventions for ADHD could benefit from repetition and training, a strategy that is currently overlooked [24,25].

Importantly, performance improvement from the pre-sleep to the post-sleep test was influenced by the interaction of repeated task exposure and sleep; sufficient sleep allowed greater performance improvement in dogs with higher ADHD scores. In dogs, the beneficial effect of sleep in declarative memory tasks such as command learning has been shown [41,42,43], and our findings suggest that it may also support improvements in cognitive flexibility in a spatial reversal learning context. In humans, although short-term practice effects on cognitive flexibility have been reported in adolescents using the Wisconsin Card Sorting Test [23], their relevance in relation to ADHD has barely been systematically investigated, to the best of our knowledge.

Supporting our findings and highlighting the role of sleep, sleep deprivation has been shown to impair reversal learning performance in humans [68]. We propose that repeated task exposure and sufficient sleep may improve cognitive flexibility in dogs with ADHD-like traits. While the large variability in sleep efficiency across subjects provided detailed insight into the potential role of sleep, conclusive interpretation of sleep-dependent memory consolidation would require a no-sleep control group that remains awake and engages in other activities such as walking, playing, or chewing between tests, given that these types of post-learning behaviours may facilitate memory consolidation in dogs [42,69]. As this comparison was beyond the scope of the current study, further research is needed to explore the specific mechanisms, limitations, and generalisability of the observed improvements, as well as the distinct contribution of sleep.

We found no association with age in the pre-sleep test; however, in the post-sleep test, older dogs showed weaker reversal learning performance, aligning with previous findings [18,19,20,21]. This discrepancy may be explained by the fact that our sample intentionally included some dogs with high ADHD scores, who are most probably underrepresented in prior studies, due to the positively skewed distribution in typical convenience samples [33]. Their pronounced impairments may have initially masked age-related effects, which re-emerged once ADHD-related effects diminished by the second, post-sleep test.

The challenges associated with using non-invasive sleep EEG in dogs with more pronounced ADHD-like traits were evident both in the longer electrode attachment procedure and in the reduced success of data collection. In contrast, the difficulties faced with younger dogs during electrode application could eventually be overcome and did not result in data loss. This canine sleep EEG methodology has been applied successfully in numerous studies without requiring any pre-training of adult [70,71] and juvenile [72,73] dogs and even hand-raised wolves [74] and cats [75]. Participant exclusions (if mentioned) in these studies were generally due to specific experimental criteria, e.g., insufficient time spent in a particular sleep stage, or technical issues like excessive muscle-related artifacts [76,77]. Considering the authors’ extensive hands-on experience using this method with many dogs and the relatively low success rate observed in the current study, these findings carry important information for future research. Dogs with pronounced ADHD-like traits may require more familiarisation with the experimenter, the sleep laboratory, and the electrodes to ensure successful sleep EEG data collection. Otherwise, these dogs are more likely to be excluded due to measurement failure, potentially reducing sample representativeness, leading to biased or non-generalisable conclusions. Incorporating multiple familiarisation sessions or improvements in methodology (e.g., using wireless electrodes) may help overcome these challenges.

A limitation of our study was the different composition of the sample from that used in human research. Instead of investigating differential associations in clinical and non-clinical populations, we examined associations with the total ADHD score as a continuous variable. This approach was necessary, as validated diagnostic criteria for canine ADHD were only recently introduced and were not available at the time of data collection [35]. Nevertheless, our findings remain valuable, as recent views in the human literature increasingly support a neurodevelopmental spectrum in transdiagnostic research, rather than relying on dichotomous diagnostic classifications [78]. Second, although we aimed to minimise variability in between-test intervals, the success and speed of the electrode attachment procedure inevitably varied to some degree. Nevertheless, all dogs were provided a minimum one-hour-long break before the second reversal task. It remains a question for future research whether sleep specifically contributes to the observed effects, or whether other forms of quiet rest may be sufficient. Our findings raise the possibility that resting (thus calming down to be able to sleep) may need to be trained in dogs with ADHD-like traits, similarly to children with ADHD [79]. Lastly, the complexity of our design presents both a limitation and a strength: while simplified experimental setups are better suited for isolating individual effects, only complex designs can capture the interrelated nature of multifaceted phenomena.

## 5. Conclusions

We demonstrated that family dogs’ cognitive flexibility is associated with their ADHD-like traits and sleep patterns. Specifically, repeated task exposure combined with sufficient sleep between testing may help mitigate reversal learning impairments linked to ADHD symptoms. The feasibility of the sleep EEG measurement was influenced by the dogs’ ADHD-like traits, with less success in higher-scoring dogs. An additional contribution of this study involves drawing attention to the methodological challenges of using non-invasive sleep EEG in dogs with more pronounced ADHD-like traits. These difficulties carry important implications for future research, underscoring the need for cautious interpretation of results that may be influenced by sampling bias. Further, improving EEG methodologies could enable data collection in dogs with stronger ADHD-like traits. Such advancements could help in identifying the neural mechanisms underlying ADHD-related phenomena. Our findings contribute to a deeper understanding of canine executive functioning and may inform future therapeutic strategies for managing ADHD-related impairments in dogs and, in the long term, potentially in humans.

## Data Availability

The raw data supporting the conclusions of this article will be made available by the authors on request.

## Supplementary Materials

The following supporting information can be downloaded at: https://www.mdpi.com/article/10.3390/ani15213074/s1, Figure S1: The distribution of total Attention-Deficit/Hyperactivity Disorder (ADHD) scores in the N = 58 sample; Table S1: Results of the GLMs; Table S2: Data of the subjects passing and failing different test phases; Table S3: Results of the Cox proportional hazards model. Reference [33] is cited in the Supplementary Materials.
