# Supplementary material for: Repeated Task Exposure and Sufficient Sleep May Mitigate ADHD-Related Cognitive Flexibility Impairments in Family Dogs
Source: Animals (Basel). 2025 Oct 23;15(21):3074. doi: 10.3390/ani15213074 (PMC12609662; doi:10.3390/ani15213074)
Supplement: Supplementary file 1 [file animals-15-03074-s001.zip › animals-3919962-supplementary.pdf]

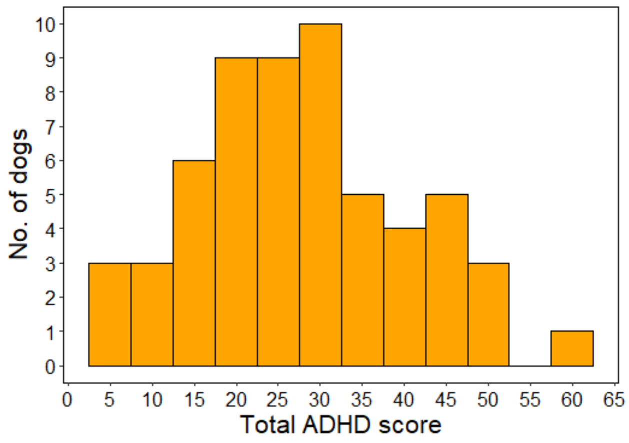

Figure S1: The distribution of total Attention-Deficit/Hyperactivity Disorder (ADHD) scores in the N=58 sample. For reference of a typical positively skewed distribution in a large (N>1000) sample, see [33].

Dependent variable	N	Term	Estimate	SE	z-value / t-value	p
r1 trial count	49	(Intercept)	2.66	0.12	21.74	<0.001***
		total ADHD score	0.01	<0.01	2.75	0.006**
		training - intermediate	-0.13	0.09	-1.4	0.16
		training - advanced	-0.01	0.08	-0.09	0.932
		age	0.002	<0.01	1.33	0.181
r2 trial count	36	(Intercept)	2.79	0.15	18.75	<0.001***
		total ADHD score	-0.002	<0.01	-0.66	0.511
		training - intermediate	-0.14	0.11	-1.25	0.212
		training - advanced	-0.15	0.1	-1.52	0.128
		age	0.004	<0.01	2.13	0.033*
r2-r1 trial count difference	36	(Intercept)	-1.18	4.8	-0.25	0.808
		total ADHD score	-0.03	0.11	-0.27	0.788
		sleep efficiency	0.12	0.07	1.84	0.076
		training - intermediate	-2.41	2.66	-0.9	0.374
		training - advanced	-4.46	2.31	-1.93	*0.063*[#]
		age	0.02	0.04	0.55	0.588
		total ADHD score × sleep efficiency	-0.004	<0.01	-1.92	0.064[#]
sleep EEG success	46	(Intercept)	1.55	1.36	1.14	0.257
		total ADHD score	-0.07	0.03	-2.3	0.021*
		training - intermediate	1.51	1.03	1.46	0.146
		training - advanced	-0.02	0.86	-0.02	0.984
		age	0.02	0.02	1.08	0.282

Table S1: Results of the GLMs. Training level was included as a three-level factor (basic, intermediate, advanced), with 'basic' as the reference level. For the models of r1 trial count, r2 trial count, and sleep EEG success, z-values are shown, while t-values are shown for the model of r2-r1 trial count difference. *** p<0.001, ** p<0.01, * p<0.05, [#] p<0.07. *Post-hoc analysis revealed no difference between training level groups.*

<table>
<tr><th colspan="2">phase</th><th>success</th><th>N</th><th>age (M ± SD)</th><th>ADHD (M ± SD)</th></tr>
<tr><td rowspan="4">pre-sleep</td><td rowspan="2">discrimination (d1)</td><td>passed</td><td>55</td><td>43.8 ± 26.2</td><td>27.6 ± 12.7</td></tr>
<tr><td>failed</td><td>3</td><td>35 ± 12.5</td><td>40.7 ± 11.1</td></tr>
<tr><td rowspan="2">reversal (r1)</td><td>passed</td><td>49</td><td>44.2 ± 25.5</td><td>27.8 ± 13.1</td></tr>
<tr><td>failed</td><td>6</td><td>41 ± 34.1</td><td>26 ± 10.2</td></tr>
<tr><td rowspan="4">post-sleep</td><td rowspan="2">discrimination (d2)</td><td>passed</td><td>39</td><td>44.9 ± 26.1</td><td>27.3 ± 13.6</td></tr>
<tr><td>failed</td><td>0</td><td>-</td><td>-</td></tr>
<tr><td rowspan="2">reversal (r2)</td><td>passed</td><td>36</td><td>43.5 ± 24.9</td><td>27.2 ± 13.9</td></tr>
<tr><td>failed</td><td>1</td><td>15</td><td>43</td></tr>
</table>

Table S2: Data of the subjects passing and failing different test phases. Mean and standard deviation values are shown for age and the total ADHD score. Age is shown in months. The drop in the sample size from phase r1 to d2 was due to some owners' limited availability. Two subjects that passed phase d2 were not tested in phase r2 due to experimenter error.

Term	Coefficient	Hazard ratio	SE	z-value	p
total ADHD score	-0.06	0.94	0.02	-3.18	0.001**
training - intermediate	0.24	1.27	0.49	0.48	0.63
training - advanced	0.03	1.03	0.45	0.07	0.945
age	0.02	1.02	0.01	2.18	0.029*

Table S3: Results of the Cox proportional hazards model. The dependent variable was the time needed to attach the electrodes, the sample size was N=46. Training level was included as a three-level factor (basic, intermediate, advanced), with 'basic' as the reference level. ** $p<0.01$, * $p<0.05$.

Reference:

33. Csibra, B.; Bunford, N.; Gácsi, M. Development of a Human-Analogue, 3-Symptom Domain Dog ADHD and Functionality Rating Scale (DAFRS). *Sci Rep* **2024**, *14*, 1808, doi:10.1038/s41598-024-51924-9.
